# Supplementary material for: Snf1 Kinase Differentially Regulates Botrytis cinerea Pathogenicity according to the Plant Host
Source: Microorganisms. 2022 Feb 15;10(2):444. doi: 10.3390/microorganisms10020444 (PMC8877277; doi:10.3390/microorganisms10020444)
Supplement: Supplementary file 1 [file microorganisms-10-00444-s001.zip › microorganisms-1532303-supplementary.pdf]

Name	Sequence	Melting temperature (ºC)	Product size (bp)
Snf1-5'-For<br>Snf1-5'-Rev	5' TAGTACCTGGCGCTTCTGTG 3'<br>5' CCGGCCCGAATCGGGAAAATGTGGTTGAACGGTGGTT 3'	66	1182
Snf1-3'-For<br>Snf1-3'-Rev	5' CGTCCGAGGGCAAAGGAATAGGTTCGTTGGTTATTGAATCG 3'<br>5' CAACCAAAGAATGAAGTTCTCAC 3'	65	859
Hyg-For<br>Hyg-Rev	5' TTCCCGATTCGGGCCGG 3'<br>5' CTATTCCTTTGCCCTCGGAC 3'	60	1848
Snf1-5'-For<br>Hyg-Nest-Rev	5' TAGTACCTGGCGCTTCTGTG 3'<br>5' GGATGCCTCCGCTCGAAGTA 3'	66	2750
Hyg-Nest-For<br>Snf1-3'-Rev	5' CAG CGA GAG CCT GAC CTA TTG 3'<br>5' CAACCAAAGAATGAAGTTCTCAC 3'	65	1617
Snf1-M-5'-For<br>Snf1-M-5'-Rev	5' GCGTGGGATCGAGTTATTGC 3'<br>3' GCCCTGACTACCTTGCTACGG 5'	64	1719
Snf1-M-3'-For<br>Snf1-M-3'-Rev	5' CAATGTCCTGACGGACAATG 3'<br>5' GATTCGGATTTCTTTTGATGAGG 3'	61	1438
Snf1-WT-For<br>Snf1-WT-Rev	5' GCATTTCAGCCTCGACCTAGC 3'<br>5' CCCATGGATCTTCTGGCAAC 3'	64	643
Hyg-Probe-For<br>Hyg-Probe-Rev	5' CAAGCTGCATCATCGAAATTGC 3'<br>5' ATCGAAAAGTCCGACAGCGTC 3'	55	818
Snf1-Comp-For<br>Snf1-Comp-Rev	5' CCATGATGCTAGGGGAGG 3'<br>5' GATGATATTGGCGACCCG 3'	68	4408
Nourseo-For<br>Nourseo-Rev	5' GAGCGGATTCCTCAGTCTC 3'<br>5' GAGTGAGCTGATACCGCTCG 3'	69	2028
Snf1-5'-For<br>Snf1-WT-Rev	5' TAGTACCTGGCGCTTCTGTG 3'<br>5' CCCATGGATCTTCTGGCAAC 3'	60	3296
Snf1-WT-For<br>Snf1-3'-Rev	5' GCATTTCAGCCTCGACCTAGC 3'<br>5' CAACCAAAGAATGAAGTTCTCAC 3'	59	1845
Bc-Tub-For<br>Bc-Tub-Rev	5' GTCTCAAGATGTCCTCCACC 3'<br>5' ACTCCATCTCGTCCATACCT 3'	62	143
Snf1-ORF-For<br>Snf1-ORF-Rev	5' CGAACCATCACCTCAAACAGAC 3'<br>5' ATCATTGCGATTATTCCCGTTACC 3'	62	291

Supplementary Table S1: Sequences of the primers pairs used in this study
